# Supplementary material for: MST2 kinase suppresses rDNA transcription in response to DNA damage by phosphorylating nucleolar histone H2B
Source: EMBO J. 2018 May 22;37(15):e98760. doi: 10.15252/embj.201798760 (PMC6068430; doi:10.15252/embj.201798760)
Supplement: Supplementary file 1 — Expanded View Figures PDF [file EMBJ-37-e98760-s001.pdf]

Expanded View Figures

Figure EV1. Phosphorylation of histone H2B at serine 14 accumulates in the nucleolus in response to DNA damage.

A U2OS cells were treated or not with 50 μM etoposide overnight. Total cell extracts were analysed by Western blot for the indicated antibodies.
B U2OS cells were transfected with pcDNA3.1, H2B-GFP or H2BS14A-GFP and treated with 50 μM etoposide overnight. Total cell extracts were analysed by Western blot for the indicated antibodies (ab_1 H2BS14p from Cell Signalling, ab_2 H2BS14 from Millipore).
C H2BS14p staining in etoposide-induced apoptosis (left), mitotic chromatin (middle) or midbody during telophase (right).
D HeLa cells were exposed to 5 Gy γIR, fixed at the indicated time points and stained for H2BS14p (Cell signalling) and nucleolin. Selected areas are shown in higher magnification in Fig 1C.
E HeLa cells were treated with γIR and stained for H2BS14p (Millipore) at the indicated time points.
F U2OS cells were treated with γIR fixed and stained for H2BS14p and nucleolin at the indicated times.
G HBECS cells were irradiated and fixed 10 min after exposure. Control and irradiated cells were stained with the indicated antibodies.

Data information: DNA was stained with DAPI. Scale bars at 10 μm.
Source data are available online for this figure.

© 2018 The Authors

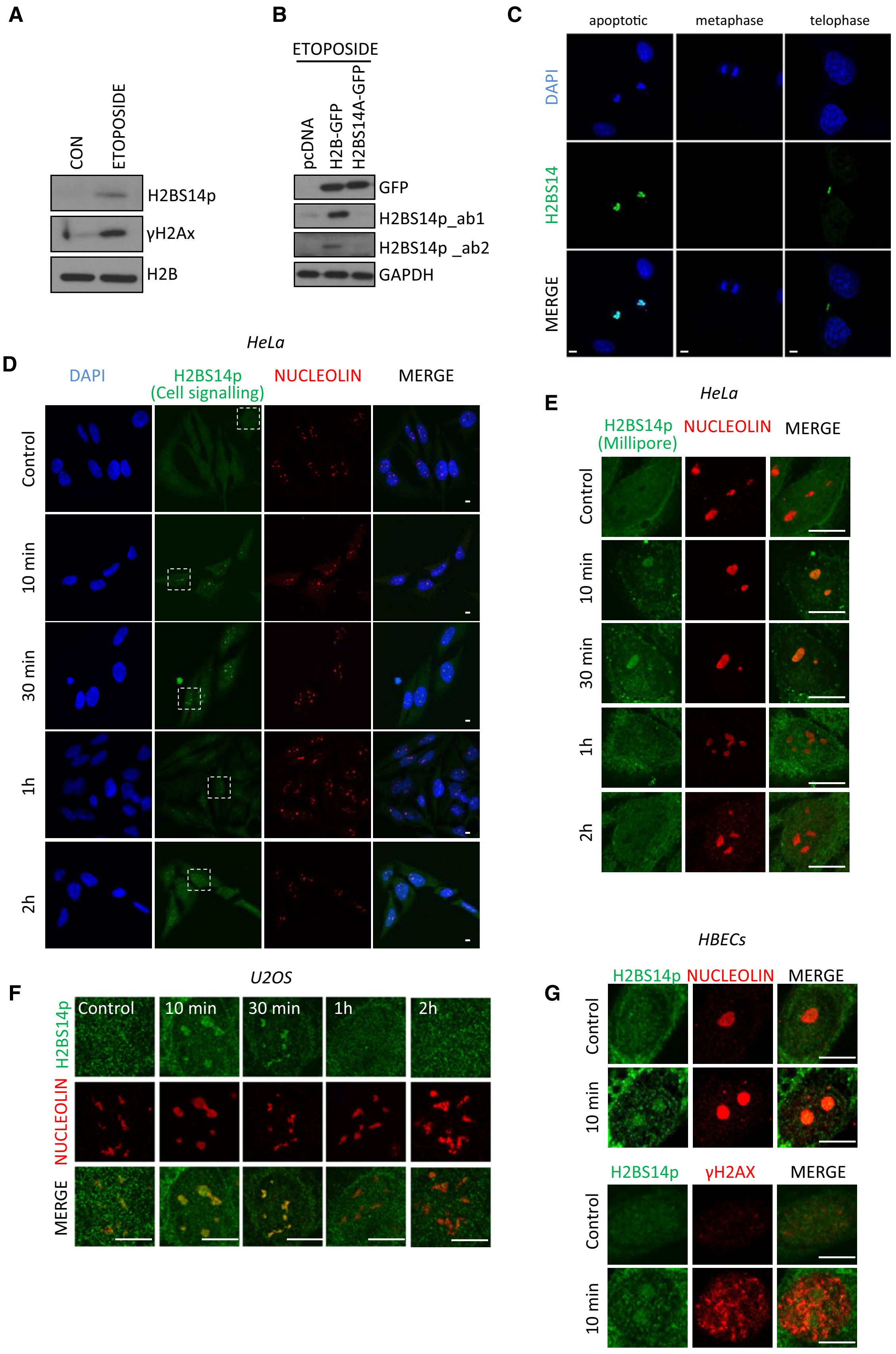

Figure EV1.

© 2018 The Authors

Figure EV2. MST2 kinase targets nucleolar H2BS14p to regulate rDNA transcription.

A U2OS Cells were treated siRNA against MST2 or control siRNA, fixed and stained for MST2.

B HeLa cells were exposed to γIR, lysed, and anti-MST2 or IgG control antibody was used to immunoprecipitate proteins from total cell extracts prior to Western blotting with indicated antibodies.

C U2OS cells were treated with the indicated siRNAs, exposed to γIR, fixed and stained with the indicated antibodies.

D HeLa cells were treated with siLUC or siMST2 exposed to γIR, collected at the indicated time points and stained for the indicated antibodies.

E HeLa cells were treated or not with the ATM inhibitor, exposed to γIR and treated with 0.5 mM 5-EU for 20 min. 5-EU incorporation was quantified and representative images from each condition are shown. Data derive from two independent experiments. Middle line represents the median and the boxes 25th and 75th percentiles. The whiskers mark the smallest and largest values. The Mann–Whitney test was used for statistical analysis. ***$P < 0.001$.

F Representative images from HeLa cells treated with the indicated siRNAs, exposed to γIR and treated with 0.5 mM 5-EU for 20 min. Quantification is shown in Fig 4C.

G HeLa cells were treated with a second RNAi oligo that targets MST2 mRNA (siMST2_2) or control siRNA. Western blot analysis was used to assess MST2 levels 48 h after transfection.

H HeLa cells were treated with the indicated siRNAs, exposed to γIR, fixed after 10 min and stained for the indicated antibodies. DNA was stained with DAPI.

I HeLa cells were treated with the indicated siRNAs, exposed to γIR and treated with 0.5 mM 5-EU for 20 min. Quantification of 5-EU nuclear intensity in each condition is shown. Data derive from two independent experiments, and the graph represents data as described in (E). ***$P < 0.001$.

J HeLa cells were treated with siMST2_2 and exposed to γIR. 20 min after exposure pre-rRNA levels were assessed using qPCR. Error bars indicate SD and derive from two independent experiments. **$P < 0.01$.

Data information: Scale bars at 10 μm. Two-tailed Student's *t*-test was used for statistical analysis.
Source data are available online for this figure.

© 2018 The Authors

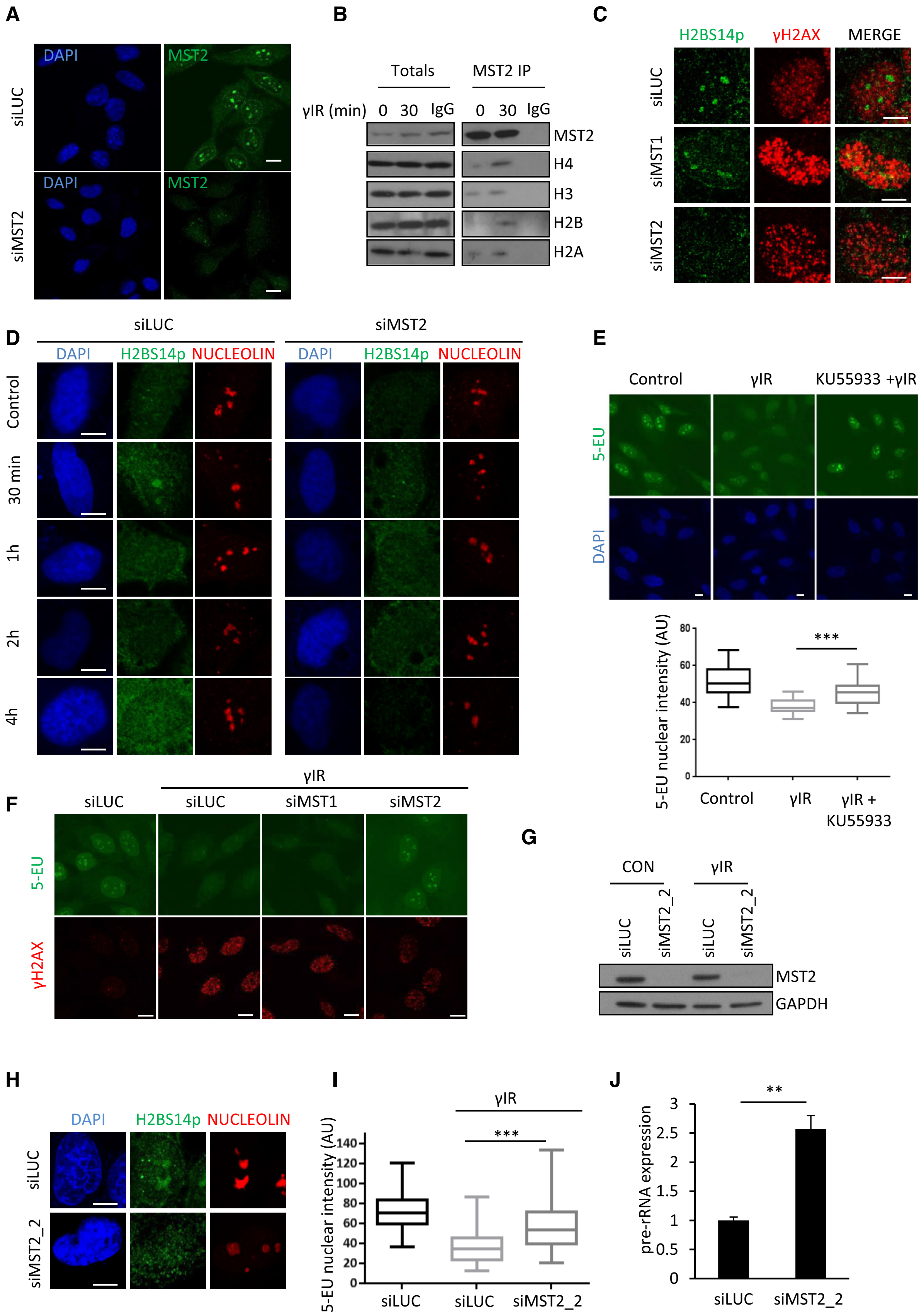

Figure EV2.

© 2018 The Authors

Figure EV3. ATM-RASSF1A-MST2 axis is necessary for H2BS14p establishment in response to DNA damage.

A Expression of H2B-GFP, H2BS14A-GFP and H2BS14D-GFP in HeLa cells.
B U2OS cells were transfected with H2B-GFP or H2BS14D-GFP. Relative pre-rRNA expression, normalised against GAPDH was assessed with qPCR. Error bars represent the SD and derive from two independent experiments.
C HeLa cells were transfected with H2B-GFP or H2BS14D-GFP. Relative pre-rRNA expression, normalised against beta-2-microglobulin was assessed with qPCR. Error bars represent the SD and derive from two independent experiments.
D HeLa cells were transfected with H2B-GFP or H2BS14A-GFP and exposed to γIR. 20 min after induction of DNA damage pre-rRNA expression normalised against beta-2-microglobulin was assessed with qPCR. Error bars represent the SD and derive from two independent experiments.
E U2OS cells were treated with γIR or with the RNA Pol I inhibitor CX-5461, fixed and stained for the indicated antibodies.
F HeLa cells were treated with DMSO, KU55933 (ATM inhibitor), NU7441 (DNA-PK inhibitor) or olaparib (PARP inhibitor) and exposed to γIR. Cells lysates were analysed with Western blot for the indicated antibodies.
G HeLa cells were treated with the indicated siRNAs exposed to γIR and fixed or lysed. Fixed cells were stained with the indicated antibodies (left), and cell lysates were analysed with Western blot for the indicated antibodies (right).

Data information: Scale bars at 10 μm. Two-tailed Student's *t*-test was used for statistical analysis. $^{**}P < 0.01$, $^{***}P < 0.001$.
Source data are available online for this figure.

© 2018 The Authors

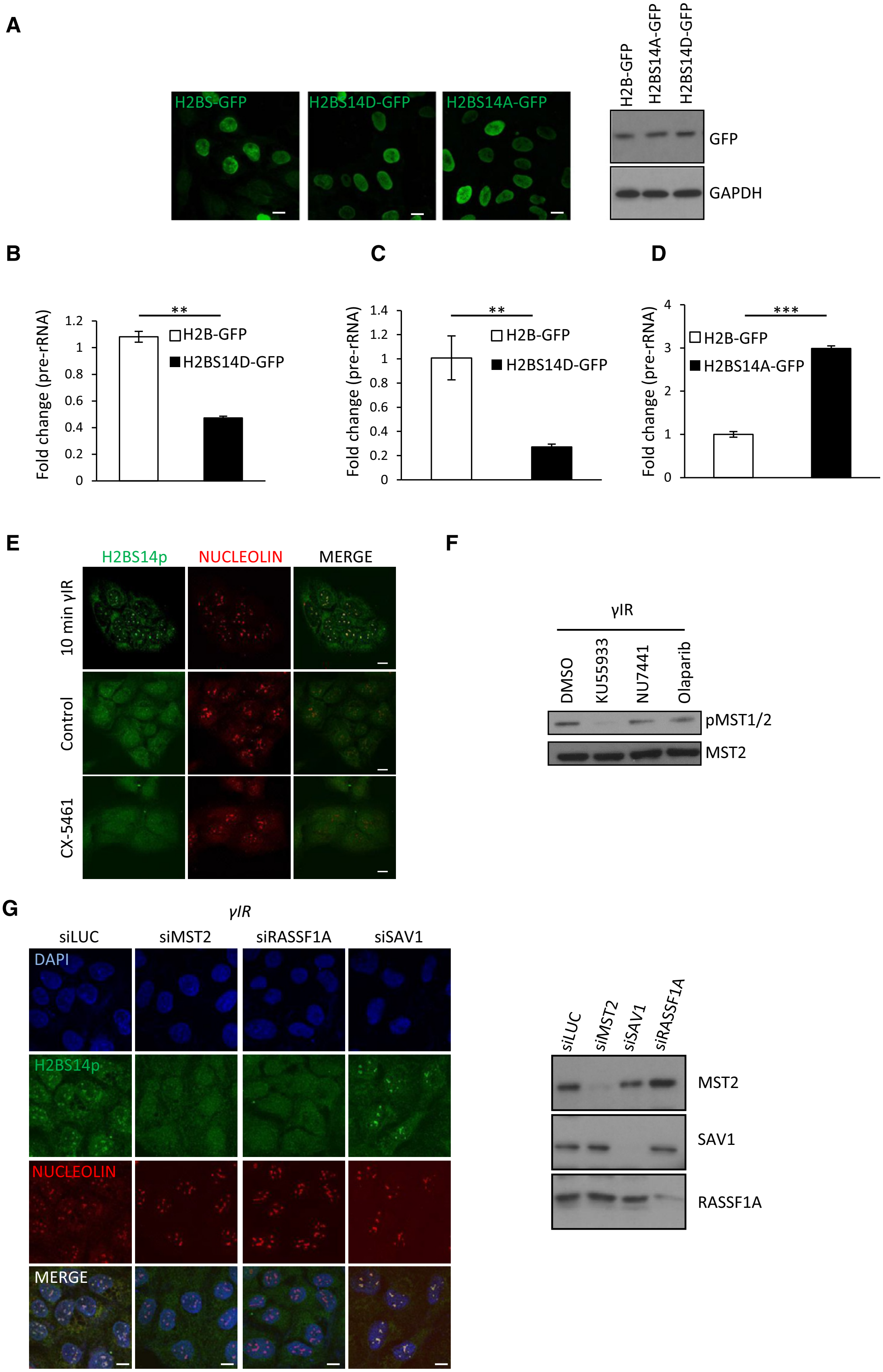

Figure EV3.

 © 2018 The Authors

Figure EV4. MST2 regulates rDNA transcription in response to rDNA DSBs.

A HeLa cells were transfected with *in vitro* transcribed I-PpoI WT or I-PpoI H98A mRNA and were stained for UBF 6 h post-transfection. UBF marks nucleolar caps in I-PpoI WT damaged nucleoli.

B HeLa cells were transfected with I-PpoI WT in the presence of 10 μM KU55933 or DMSO, and 5-EU incorporation was assessed by immunofluorescence 6 h post-mRNA transfections. Representative images and quantification of V5-positive cells that incorporate 5-EU are shown. Error bars representing the SD derived from two independent experiments.

C HeLa cells were transfected with V5-I-PpoI WT mRNA and treated with 5-EU for 20 min at the indicated times. 5-EU incorporation was assessed in V5-positive cells.

D rDNA DSBs induced by I-PpoI WT mRNA in HeLa cells and H2BS14p levels determined by immunofluorescence at the indicated times. DNA was stained with DAPI.

E HeLa cells were transfected with the indicated siRNAs and transfected with I-PpoI WT mRNA. 5-EU/V5 double-positive HeLa cells were quantified, and representative images are shown. Error bars representing the SD derived from two independent experiments.

F HeLa cells were transfected with siMST2_2, and 48 h post-transfection I-PpoI WT mRNA was introduced. Representative images and quantification of V5-positive cells that incorporate 5-EU are shown. Error bars representing the SD derived from two independent experiments.

G HeLa cells were transfected with H2B-GFP and H2BS14A-GFP and 24 h later transfected with I-PpoI WT mRNA. Pre-rRNA expression relative to beta-2-microglobulin was assessed with qPCR. Error bars represent the SD and derive from two independent experiments.

Data information: Scale bars at 10 μm. Two-tailed Student's t-test was used for statistical analysis. *$P < 0.05$, **$P < 0.01$, ***$P < 0.001$.

© 2018 The Authors

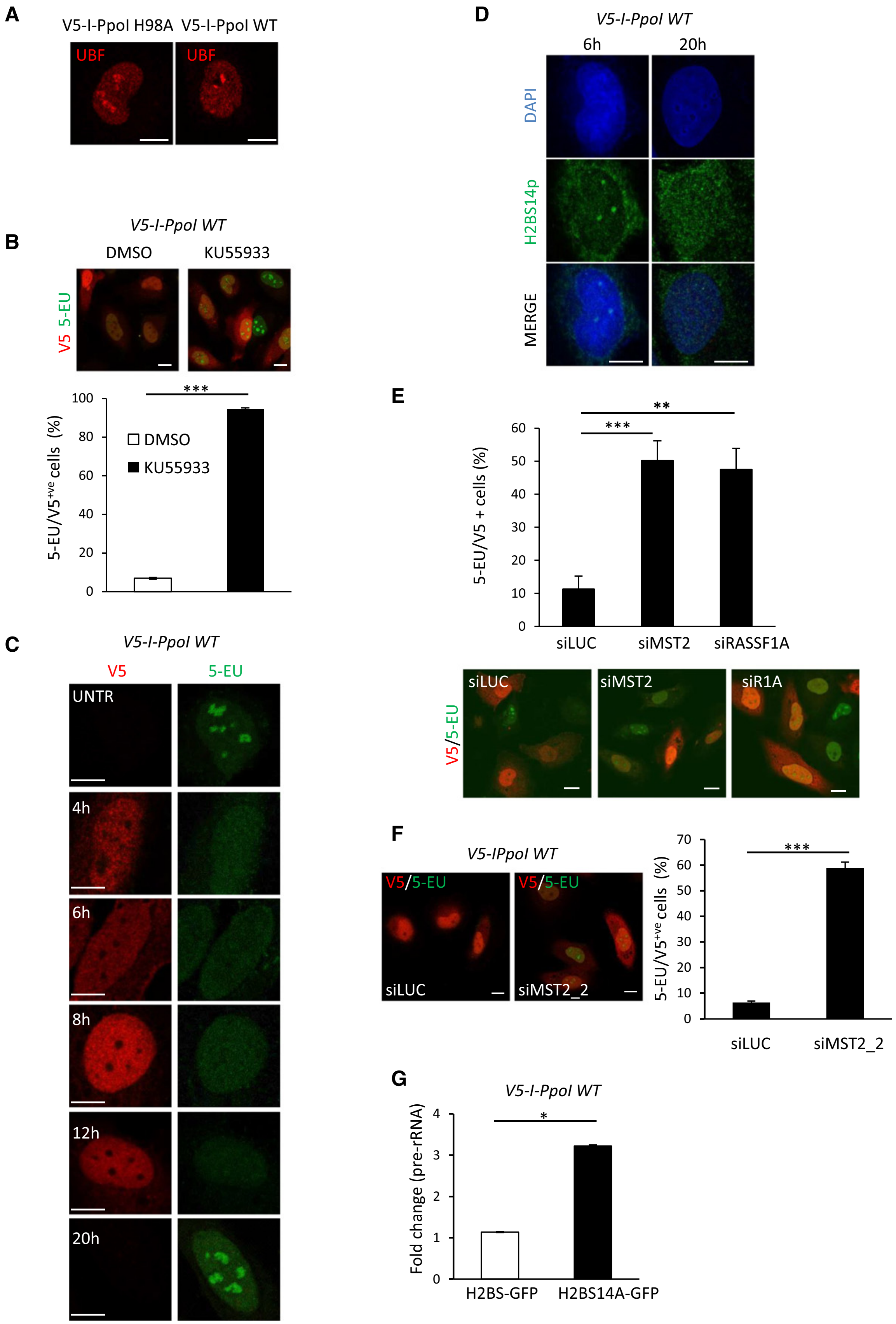

Figure EV4.

 © 2018 The Authors

Figure EV5. Phosphorylation of nucleolar H2B at serine 14 in response to rDNA DSBs.

A HeLa cells were treated with the indicated siRNAs/ATM inhibitor, and I-PpoI WT or I-PpoI H98A mRNA was introduced. Cells were fixed and stained for the indicated antibodies. Representative images (left) and quantification (right) of nucleolar segregation based on UBF staining are shown. Representative images on UBF staining used to assess nucleolar segregation are shown. Selected areas are shown in higher magnification as examples of normal, partially and fully segregated nucleoli. Error bars represent the SD and derive from three independent experiments.

B HeLa cells were transfected with I-PpoI WT mRNA, fixed after 6 h and stained for the indicated antibodies.

C Clonogenic survival assay to assess sensitivity to rDNA damage. Cells were transfected with I-PpoI WT or I-PpoI H98A mRNA. The survival ratio is presented as I-PpoI WT/I-PpoI H98A ratio in each condition. Error bars derive from two independent experiments and represent SD. Representative images from each condition are shown.

Data information: Scale bars at 10 μm. Error bars represent the SD and derive from three independent experiments. Two-tailed Student's *t*-test was used for statistical analysis. $^{**}P < 0.01$, $^{***}P < 0.001$.

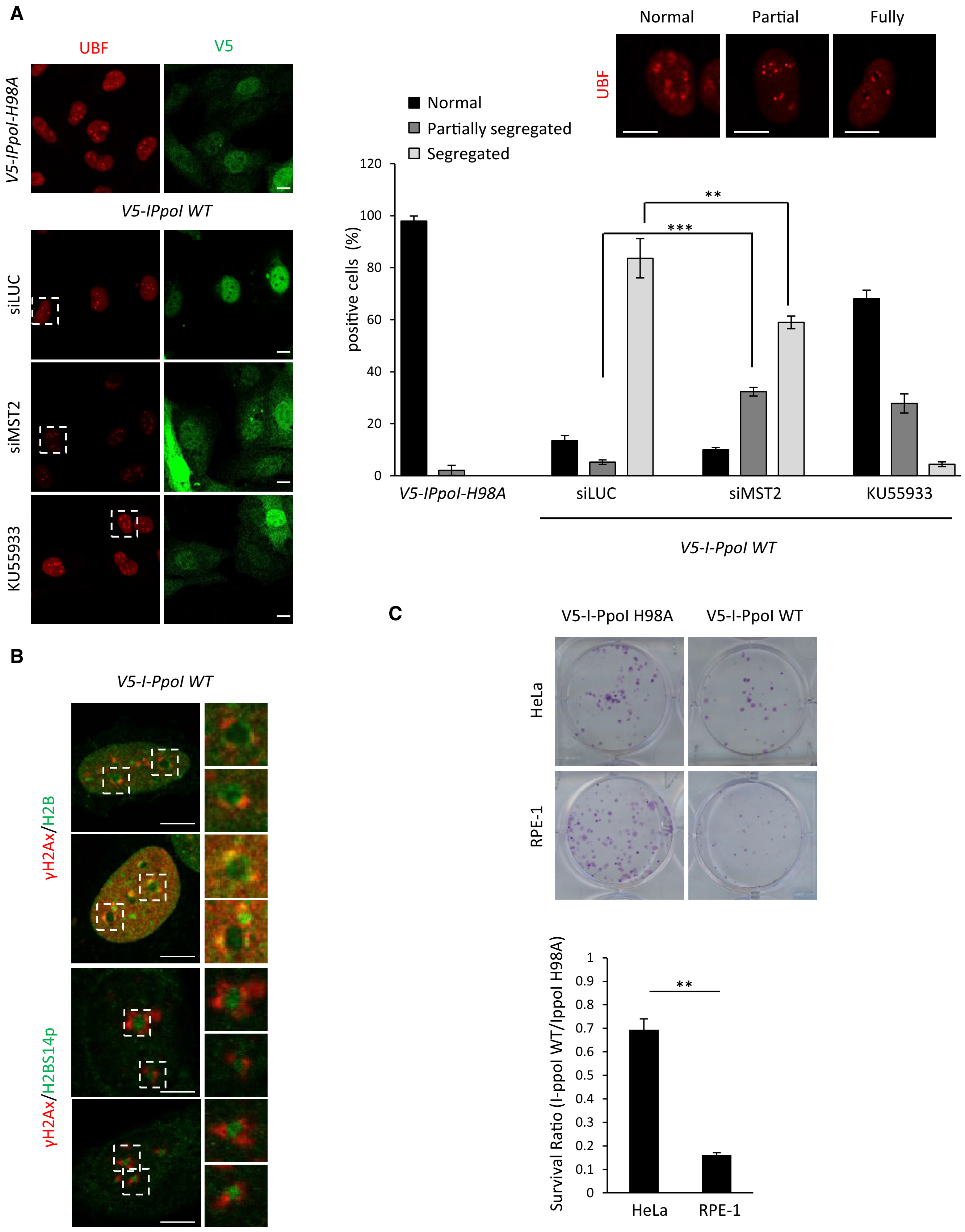

Figure EV5.

 © 2018 The Authors
