# Supplementary material for: MST2 kinase suppresses rDNA transcription in response to DNA damage by phosphorylating nucleolar histone H2B
Source: EMBO J. 2018 May 22;37(15):e98760. doi: 10.15252/embj.201798760 (PMC6068430; doi:10.15252/embj.201798760)

Figure EV1. Phosphorylation of Histone H2B at Serine 14 accumulates in the nucleolus in response to DNA damage.

EV1A

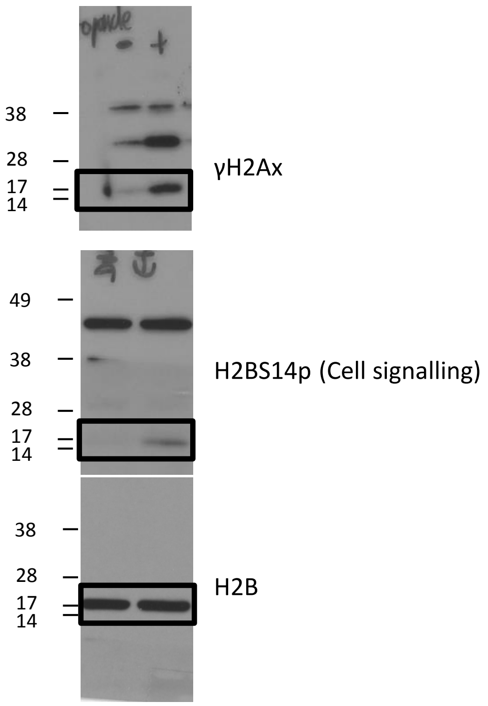

EV1B

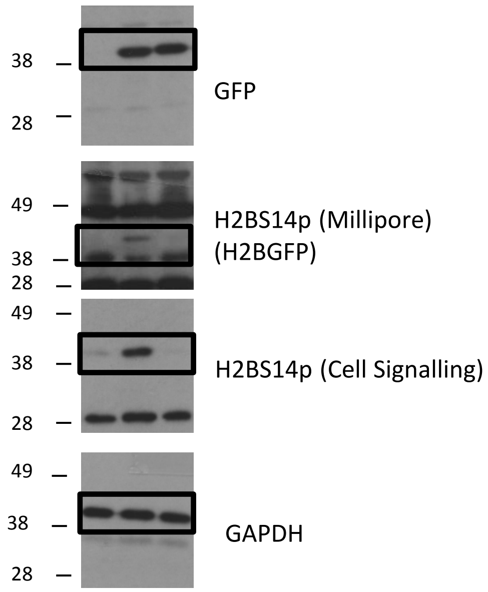

Supplement: Supplementary file 2 — Source Data for Expanded View [file EMBJ-37-e98760-s006.zip › EMBOJ_98760_source_data_for_EV1/EMBOJ_98760_source_data_for_EV1.pdf]
