# Supplementary figures and images for: MST2 kinase suppresses rDNA transcription in response to DNA damage by phosphorylating nucleolar histone H2B
Source: EMBO J. 2018 May 22;37(15):e98760. doi: 10.15252/embj.201798760 (PMC6068430; doi:10.15252/embj.201798760)

Figure EV2. MST2 kinase targets nucleolar H2BS14p to regulate rDNA transcription

EV2B

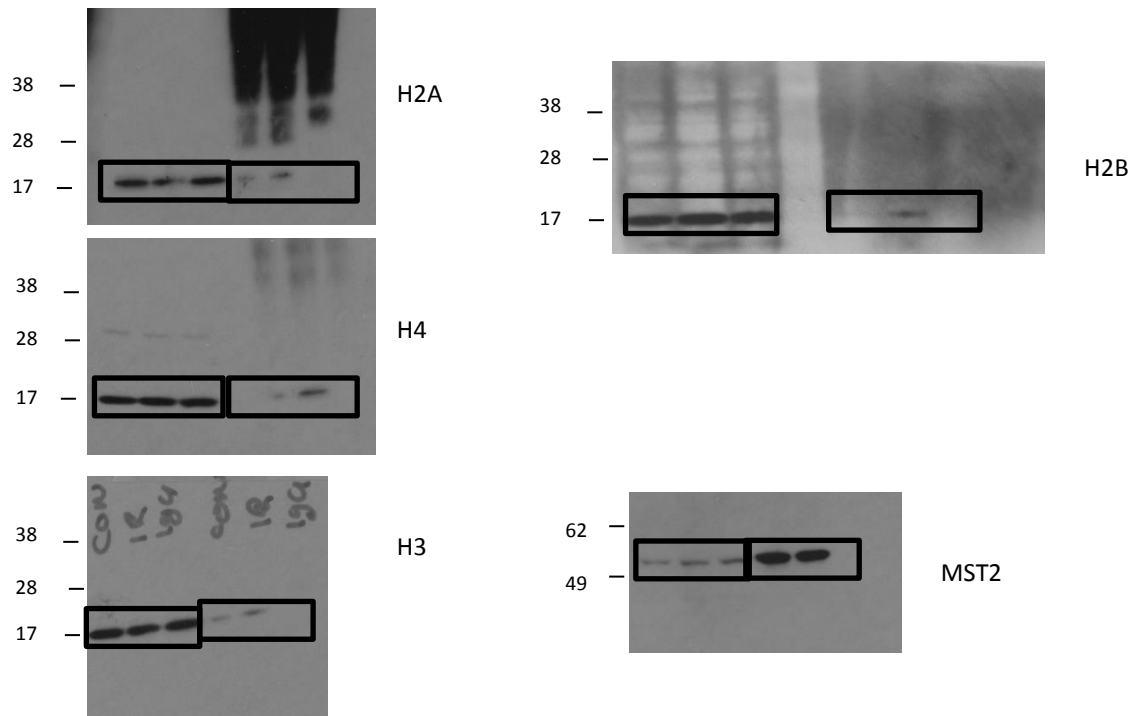

EV2G

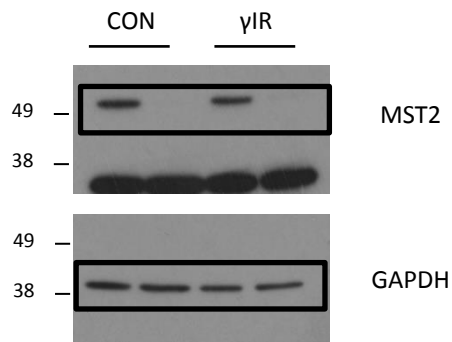

Supplement: Supplementary file 2 — Source Data for Expanded View [file EMBJ-37-e98760-s006.zip › EMBOJ_98760_source_data_for_EV2.pdf]

Figure 1: Nucleolar histone H2B gets phosphorylated at Serine 14 in response to  $\gamma$ IR

1D

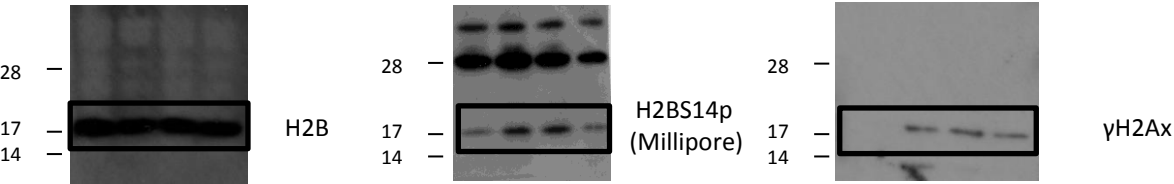

Supplement: Supplementary file 4 — Source Data for Figure 1 [file EMBJ-37-e98760-s002.pdf]

**Figure 3. MST2 phosphorylates nucleolar H2B at Serine 14 in response to  $\gamma$ IR.**

**3A**

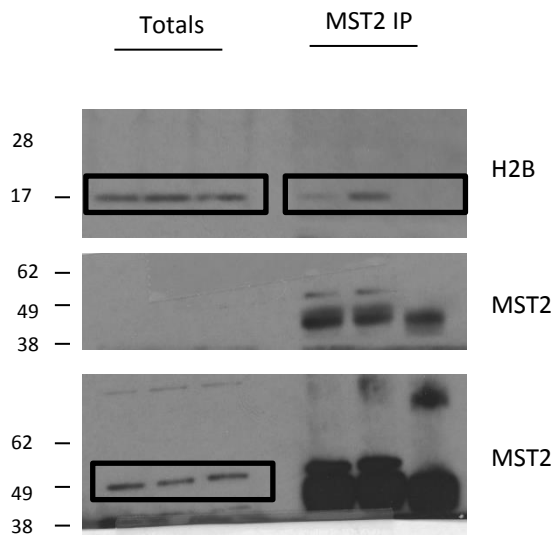

**3D**

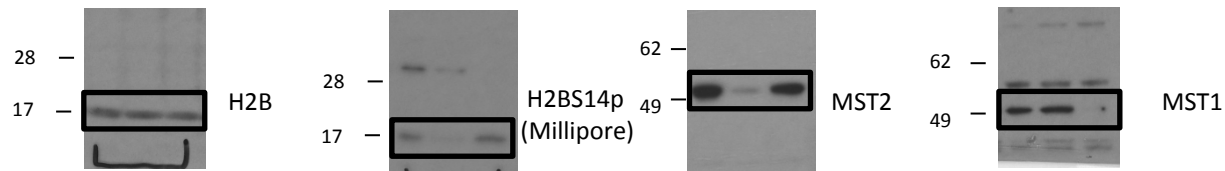

Supplement: Supplementary file 5 — Source Data for Figure 3 [file EMBJ-37-e98760-s003.pdf]

Figure 5. Nucleolar H2BS14p establishment depends on the ATM-RASSF1A-MST2 axis

5B

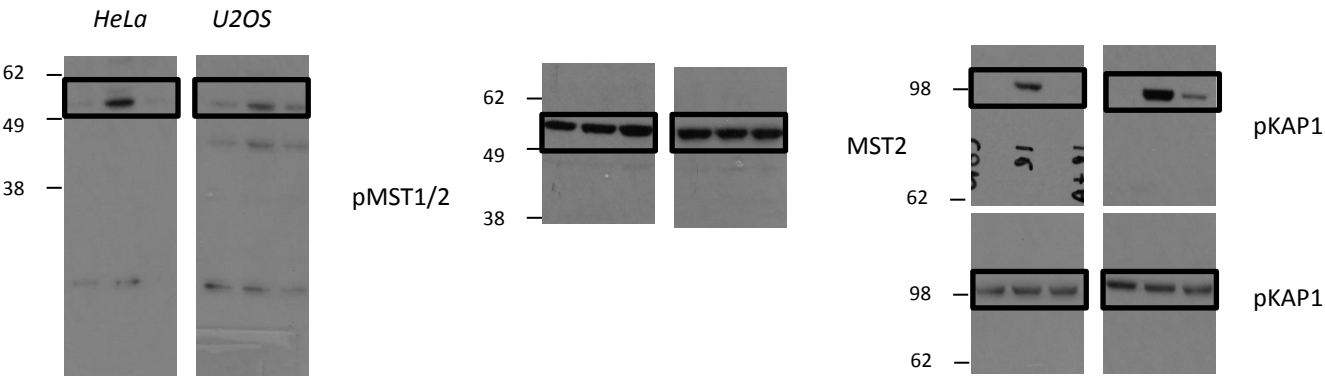

5E

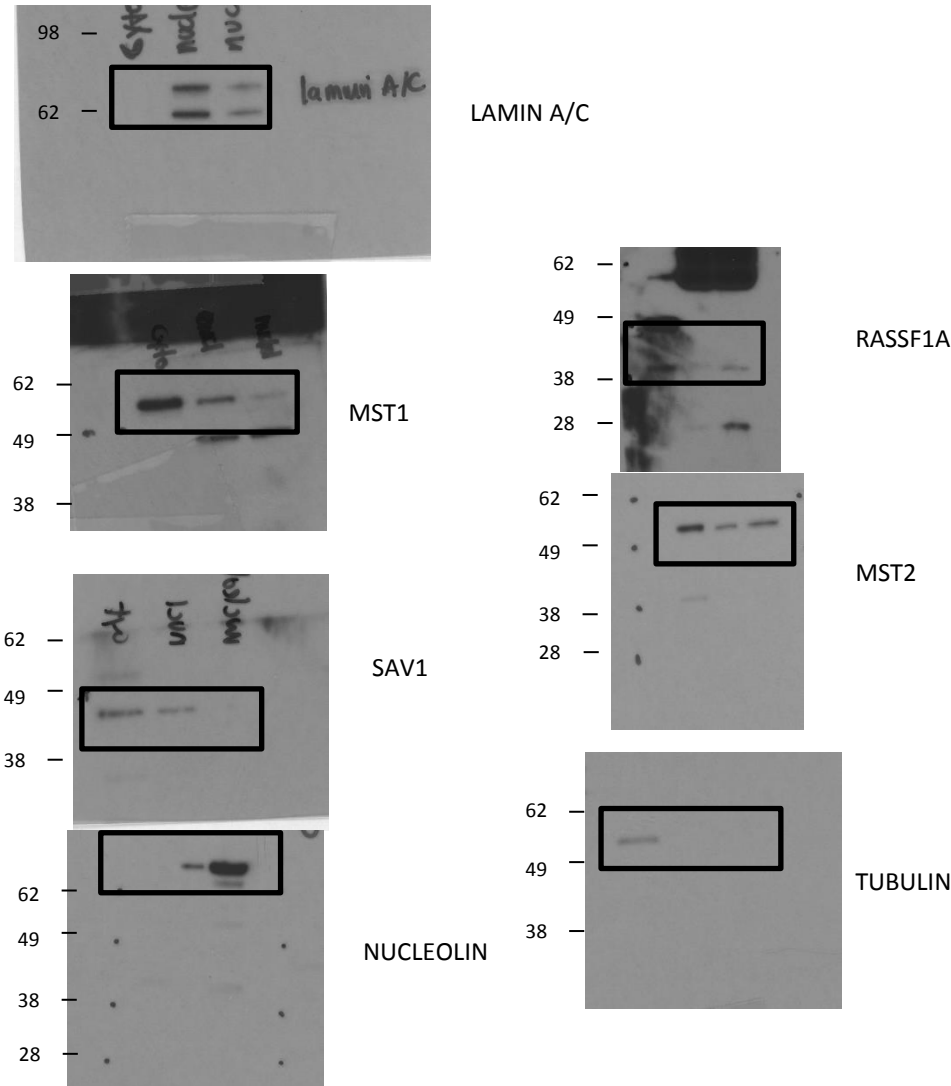

Supplement: Supplementary file 6 — Source Data for Figure 5 [file EMBJ-37-e98760-s004.pdf]

Figure 6. rDNA DSBs result in MST2 dependent transcriptional shut down.

6A

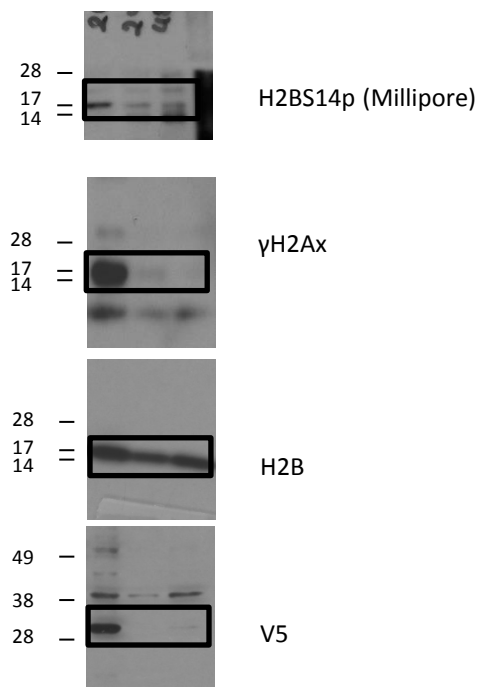

Supplement: Supplementary file 7 — Source Data for Figure 6 [file EMBJ-37-e98760-s005.pdf]
